# Effects of repeated comparative intradermal tuberculin testing on test results: a longitudinal study in TB-free red deer

**DOI:** 10.1186/s12917-016-0825-2

**Published:** 2016-09-05

**Authors:** Azlan Che-Amat, Maria Ángeles Risalde, David González-Barrio, Jose Antonio Ortíz, Christian Gortázar

**Affiliations:** 1 SaBio, Instituto de Investigación en Recursos Cinegéticos IREC (CSIC-UCLM-JCCM), Ronda de Toledo s.n., 13005 Ciudad Real, Spain; 2 Faculty of Veterinary Medicine, Universiti Putra Malaysia, 43400 Serdang, Selangor, Malaysia; 3 Medianilla Red Deer Genetics, Benalup, Cádiz, Spain

**Keywords:** *Cervus elaphus*, Farmed deer, Immune response, mycobacterial diseases, Temporal variability

## Abstract

**Background:**

Diagnosing tuberculosis (TB) in farmed red deer (*Cervus elaphus*) is challenging and might require combining cellular and humoral diagnostic tests. Repeated skin-testing with mycobacterial purified protein derivatives (PPDs) might sensitize or desensitize the subjects to both kinds of diagnostic tools. We evaluated the effect of repeated (every 6 months) comparative tuberculin skin testing on skin test and ELISA responsiveness in farmed red deer hinds from a TB-free herd. Eighteen 8-month old hinds were inoculated with bovine and avian PPDs and the mitogen phytohaemagglutinin (PHA), as positive control and concurrently tested by ELISA for antibodies against avian (avian PPD, aPPD and protoplasmatic antigen 3, PPA3) and bovine antigens (bPPD and MPB70). Blood serum was also sampled three weeks after each skin testing round and tested for antibodies against aPPD and bPPD, in order to detect eventual antibody level boosts. Testing took place every six months from winter 2012 until winter 2015.

**Results:**

The skin test response to both PPDs peaked during the second and third test round, returning to standard values thereafter. Individual variability was particularly high at the first year and early second year testing rounds (first intradermal test and blood sampling; first winter). The antibody response to avian antigens increased through time, while no such increase was recorded for bovine antigens. The antibody boost three weeks after skin testing was more marked for avian PPD. However, there was no consistent trend in the boosting response through time.

**Conclusion:**

Repeated comparative skin testing at six month intervals did not cause progressive increments in skin test responsiveness or antibody production. Specifically, we observed no loss of the skin test response to bPPD and also no progressive loss of the boosting effect in the ELISA responses. However, we recorded increases through time in the antibody levels against avian mycobacterial antigens, possibly due to the progressive exposure to MAP or to other cross-reacting environmental mycobacteria. These findings should be taken into account in designing and interpreting TB testing schemes in farmed deer.

**Electronic supplementary material:**

The online version of this article (doi:10.1186/s12917-016-0825-2) contains supplementary material, which is available to authorized users.

## Background

The red deer (*Cervus elaphus*) is an important game species with a broad geographical range worldwide and a developed farming industry [1, 2]. Red deer are susceptible to tuberculosis (TB) due to infection with *Mycobacterium bovis* and closely related members of the *Mycobacterium tuberculosis* complex (MTC), often becoming true reservoir hosts [3]. In addition, red deer (and particularly farmed red deer) are also susceptible to *Mycobacterium avium paratuberculosis* (MAP), the causative agent of paratuberculosis (PTB) or Johne’s disease [4]. Finally, infection due to other mycobacteria can occur and sensitization to these non-tuberculous mycobacteria can cause diagnostic cross-reactions [5]. Thus, it is crucial to test deer in farms and prior to movement in order to avoid sanitary risks [6].

The tuberculin skin-test based on the intradermal inoculation of *M. bovis*–derived purified protein derivative (bovinePPD, bPPD), is regarded as the standard in-vivo diagnostic method for TB control by the World Organization for Animal Health [7]. The intradermal skin test is based on the detection of the delayed hypersensitivity reaction that appears 72 h after the inoculation of bPPD and, in the case of the comparative intradermal test, its comparison with the reaction elicited by the inoculation of aPPD. Currently there is no formal requirement of TB testing for farmed deer in Spain. However, pre-movement tests are compulsory for translocations of live deer. For such cases, the single intradermal comparative cervical tuberculin (SICCT) test is the only approved diagnostic method. The bPPD skin response is expected to be greater in deer infected with MTC members. Likewise, if the animal is infected with mycobacteria other than the MTC, greater responses to aPPD than those observed to bPPD are expected [5, 8–10]. However as PPDs comprise a complex mixture of proteins in certain situations the specificity of the test may be compromised inducing false positive reactors [11]. Moreover, in cattle, it has been documented that repeated skin testing may cause a reduction in skin test response in natural and experimental infections with *M. bovis*. This has negative consequences on the ability to detect reactors [12, 13]. Many other factors such as age, sex, season, body condition and type of management can affect the skin test responsiveness in deer compromising the sensitivity and specificity [5, 14]. In cases of advanced TB, some animals become unable to respond to any diagnostic technique that detects cell mediated immune response. Such cases can be filtered-out using the mitogen phytohaemagglutinin (PHA) on a third injection site [15, 16]. The PHA skin fold increase is independent of the mycobacterial infection status and hence not affected by the PPD skin test results [17]. The use of PHA also allows for control in the variation in the general responsiveness to intradermal injected antigens [14]. The gamma interferon test has also been used for in vivo TB diagnosis in deer [18].

The detection of humoral responses by means of serology represents an alternative for screening herds of livestock and wild animals for mycobacterial infections [19, 20]. Serological tests for humoral response detection may be used as an alternative or more often as a complementary tool for screening mycobacterial infections in deer [21]. Unfortunately, tests aiming at MTC or MAP-specific antibody detection in red deer are not always very sensitive (72.7 to 86.7 % for MTC, references: [22–26]; 50 to 91 % for MAP, references: [22, 27]) and not too specific (83.8 to 98.0 % for MTC; [23, 26]; 88 to 99.5 % for MAP; [22, 27]). Antigens used in these tests are summarized in Additional file 1. Table S1. One additional issue measuring humoral responses is that the titer of antibodies changes significantly during the infection and they are mainly produced in advanced stages of infection [28].

It is known that the inoculation of bPPD for skin testing for TB boosts the antibody responses to the MTC in *M. bovis*-infected cattle [29–32]. Hence, it has been suggested that serological tests for TB in cattle should be performed after skin tests. Recently, Casal et al. [32] demonstrated that the use of serological testing performed after skin testing, in combination with traditional skin test procedures, increased the detection likelihood of tuberculous animals within TB-infected cattle herds, as compared to skin tests alone. However, the effects of serial injections of PPDs for skin testing on the responsiveness to skin tests and on serum antibody responses, as well as the duration and quality of the antibody boosts, have not been fully evaluated in red deer.

We hypothesized that repeated (every 6 months) skin testing with aPPD and bPPD could have an effect on the skin test responsiveness or on the antibody levels against mycobacterial antigens, causing progressive changes in these responses. This study of red deer hinds from a TB-free farm aimed to, 1) evaluate the effect of multiple skin testing with aPPD and bPPD on the skin test responsiveness; and 2) to evaluate the effect of multiple skin testing with aPPD and bPPD on the antibody levels measured by ELISA (aPPD, bPPD, MPB70 and PPA3) immediately before and (for the PPDs) three weeks after each skin testing round.

## Methods

### Animals

The present study was carried out in a TB-free (no positive cases since 2003) red deer farm in southern Spain. It is a farm with a semi-intensive management scheme, with pasture-rotation and year-round food supplementation. The farm is surrounded by a double fence that limits with a red deer hunting estate of the same ownership. TB-positive deer are sporadically detected during meat inspection of hunter-harvested deer from this estate. Moreover, MTC infection in feral pigs (*Sus scrofa*) and in badgers (*Meles meles*), both present at low density in the hunting estate, cannot be excluded.

Farmed deer are handled two times per year (summer and winter) for skin-testing, measurement, blood sampling and administration of antiparasitic drugs. Study animals included 18 deer hinds born in spring 2011 (8-month old at first testing). No initial assessment of the TB status was performed before the study started. These 18 hinds were individually identified with an ear tag and remained in the farm during the study period (2011–2015). We used hinds because stags usually are sold for release at the age of 1.5 years. Handling procedures and sampling frequency were part of the farm routine and not influenced by the study. Therefore, these procedures carried out were designed to reduce stress and health risks for subjects, according to European (86/609) and Spanish laws (RD 223/1988; RD 1021/2005), and current guidelines for ethical use of animals in research [33].

### Diagnostic tests

#### Intradermal skin test

All hinds were skin-tested and sampled seven times during the study period (in winter 2012, summer 2012, winter 2013, summer 2013, winter 2014, summer 2014 and winter 2015). Animals were handled twice per skin test, at times 0 and 72 h. Deer were moved from the paddocks to the farm enclosures and then immobilized by physical restraint using a hydraulic crush. At time 0, each animal was identified, weighed and blood samples were collected. Three areas of 3 cm × 3 cm were shaved at the left side of the mid-neck with an electric shaver and skin fold thickness was measured employing a digital cutimeter (Hauptner Instrumente GmbH, Zurich, Switzerland) three different times in each area, to the closest 0.1 mm, by the same operator. Then (from cranial to caudal) 0.1 ml of avian and bovine purified protein derivative (PPD; 25,000 IU/ml; CZ Veterinaria SL, Porriño, Spain) and the plant derived mitogen phytohaemagglutinin (250 g of PHA; Sigma, Barcelona, Spain) diluted in phosphate buffered saline as positive control, were inoculated using 1-ml syringes fitted with a 25-G ½-in. needle. At time 72 h, each animal was immobilized again by physical restraint, identified, and the skin fold thickness was measured again at each injection site three different times by the same operator. Animals were considered TB reactors in the SICCT if the skin fold increase to bPPD was greater than 2 mm and more than 1 mm larger than the skin fold increase to aPPD [34, 35]. Avian reactors were defined as those with a skin fold increase to aPPD >3 mm [36]. Deer with skin fold thickness increases of less than 0.5 mm to all 3 antigens were considered unresponsive animals [17]. Clinical signs such as evident pain, necrosis or exudation were also considered as positive reactions.

#### Serological tests

Blood samples were collected from the jugular vein before inoculation with the antigens for skin tests in different seasons (winter 2012, summer 2012, winter 2013, summer 2013, winter 2014, summer 2014 and winter 2015). In order to evaluate antibody boosts in animals after antigen stimulation with aPPD and bPPD, blood samples were also obtained three weeks after selected skin testing rounds (summer 2012, winter 2013, winter 2014 and winter 2015). Sera obtained by centrifugation (3000 g for 10 min) from blood samples were tested for antibodies against bovine PPD and avian PPD, paratuberculosis protoplasmatic antigen 3 (PPA3; Allied Monitor, Fayette, MO, USA) and *M. bovis* antigen MPB70 (Lionex Diagnostics & Therapeutics GmbH, Braunschweig, Germany) using an in-house ELISA as previously described [37, 38]. Briefly, after coating the plates overnight at 4 °C with 50 μl/well of antigen solution in carbonate–bicarbonate buffer (Sigma, Barcelona, Spain), wells were washed with phosphate buffered saline (PBS) solution containing 0.05 % Tween-20 (PBST) and blocked for 1 h at room temperature with 140 μl of blocking solution (5 % skim milk in PBST). The adsorbed sera were diluted (1:10, v/v) in blocking solution and 100 μl/well was added into duplicate wells of the antigen-coated plate. After a 1 h and 30 min incubation period at 37 °C, the plates were washed three times with PBST and 100 μl/well was added (0.002 mg/ml in PBS) of protein G horseradish peroxidase conjugate (Sigma, Barcelona, Spain) and incubated at room temperature for 1 h. After three washes, 100 μl/well of substrate solution (Fast OPD, Sigma, Barcelona, Spain) was added. The reaction was stopped with 50 μl/well of H_2_SO_4_ 3 N and the optical density (OD) was measured in a spectrophotometer at 450 nm.

Deer negative and positive control sera were included in every plate in duplicate . Pooled anti-PPD–positive serum was obtained from deer previously described as *M. bovis* culture positive and negative sera obtained from an experimental facility belonging to the University of Castilla-La Mancha with no clinical history of TB and PTB and repeated negative culture results. OD values of the animals between different plates were normalized according to the values of the negative controls included in each plate.

All ELISAs were performed at the same time by two experienced researchers with no previous knowledge of which sample was being analyzed. Sample results were expressed as an ELISA percentage (E%) that was calculated using the following formula: [sample E% = (mean sample OD/2 × mean of negative control OD) × 100]. Cut-off values were defined as the ratio of the mean sample OD to the double of mean OD of the negative control. Serum samples with E% values greater than 100 were considered positive [38]. Boosting was only investigated for avian and bovine PPD.

### Data analysis

Apparent prevalence rates were calculated based on frequencies of cases over the total number of cases sampled. The Spearman correlation test was used to assess the relationship among skin and serological test results. Pairwise comparisons were used to compare the seasonal effect on skin and serological tests results. Differences between group means and correlation coefficients among skin and serological test results were considered significant at *p* < 0.05. We used the Kolmogorov–Smirnov test to assess that the data was normally distributed. All analyses were performed using IBM SPSS Statistic Processor (Version 20.0).

## Results

**Fig. 1:**
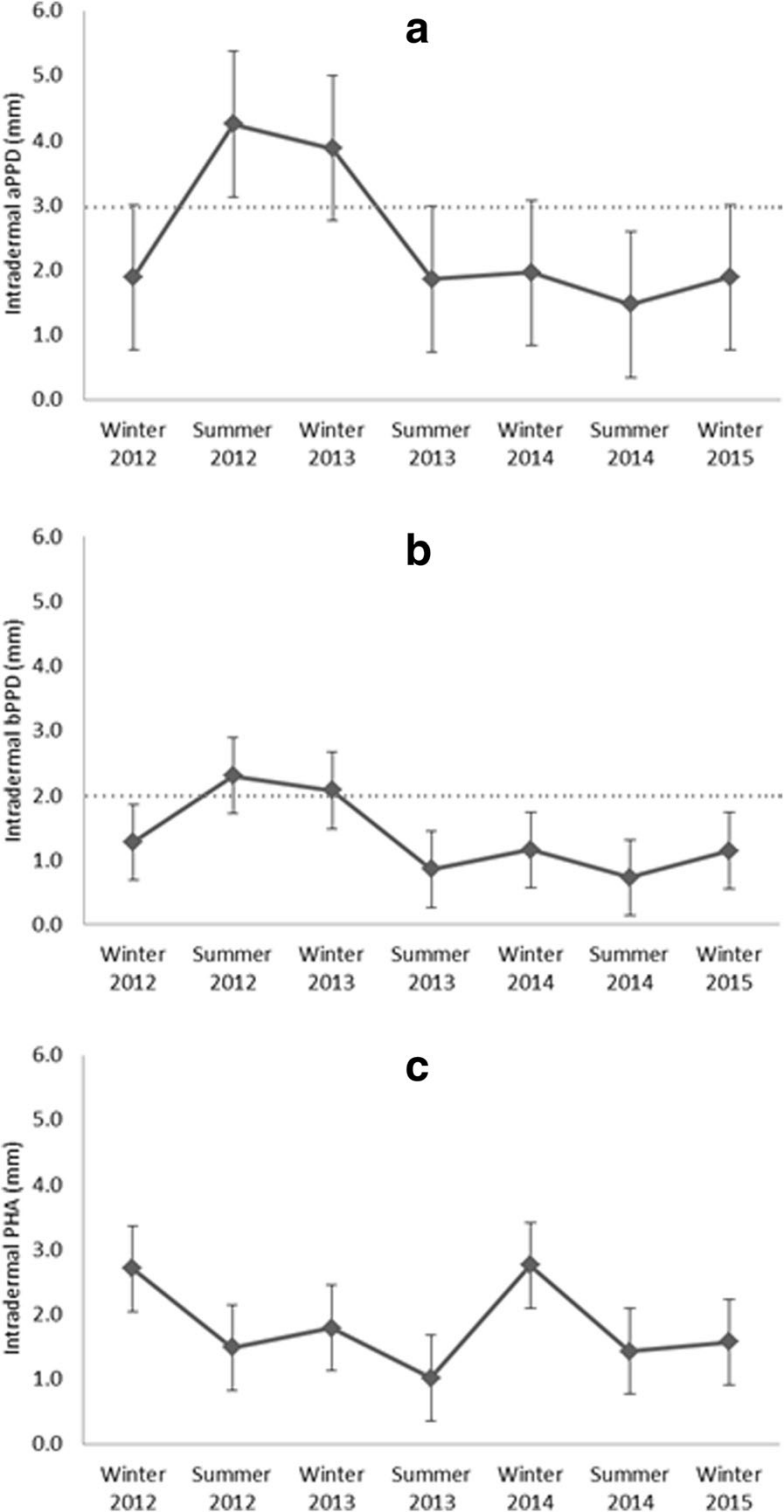
Red deer mean (±SD) seasonal (winter 2012 to winter 2015) skin test response in mm to the intradermal injection of **a** avian purified protein derivative (aPPD), **b** bovine PPD (bPPD) and **c** the mitogen phytohaemagglutinin (PHA). The dashed lines indicate the cut-off value for each PPD

**Fig. 2:**
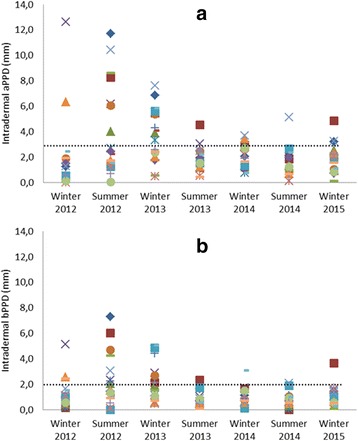
Individual seasonal (winter 2012 to winter 2015) skin test response in mm of 18 red deer hinds to the intradermal injection of **a** avian purified protein derivative (aPPD) and **b** bovine PPD (bPPD). Dashed lines indicated for cut-off value

Figure 1 shows the mean skin test responses of 18 red deer hinds for seven consecutive (six-monthly) testing rounds from winter 2012 to winter 2015. No animal was unresponsive. The mean skin test response (in mm; ±SD) was 2.42 (±2.27) to aPPD, 1.36 (±1.25) to bPPD, and 1.83 (±1.16) to PHA, respectively. The shape of the graphs for aPPD and bPPD was similar (r_s_ = 0.96; *p* < 0.05), while there was no correlation with the independent PHA (r_s_ < 0.51; n.s.). The mean skin test response to both PPDs peaked during the second and third testing round, returning to standard values thereafter, while the response to PHA had an initial peak and later a second one at the fifth testing round. Two individuals matched the definition of a reactor to the comparative skin test (responding >2 mm to bPPD and displaying a bPPD response at least 1 mm larger than aPPD). These individuals were positive in only one of seven testing rounds (round 2 and 5, respectively). However, 13 of 18 individuals (72.22 %) tested positive for the single aPPD skin test (response to aPPD >3 mm), and 7 of them tested positive in several (2 to 5) rounds. Regarding the single bPPD skin test (response to bPPD >2 mm), 13 of 18 individuals (72.22 %) tested positive for the single bPPD skin test, and 6 of them tested positive in several (2 to 4) rounds. Individual variability was high, particularly at the first year and early second year testing rounds (Fig. 2).

**Fig. 3:**
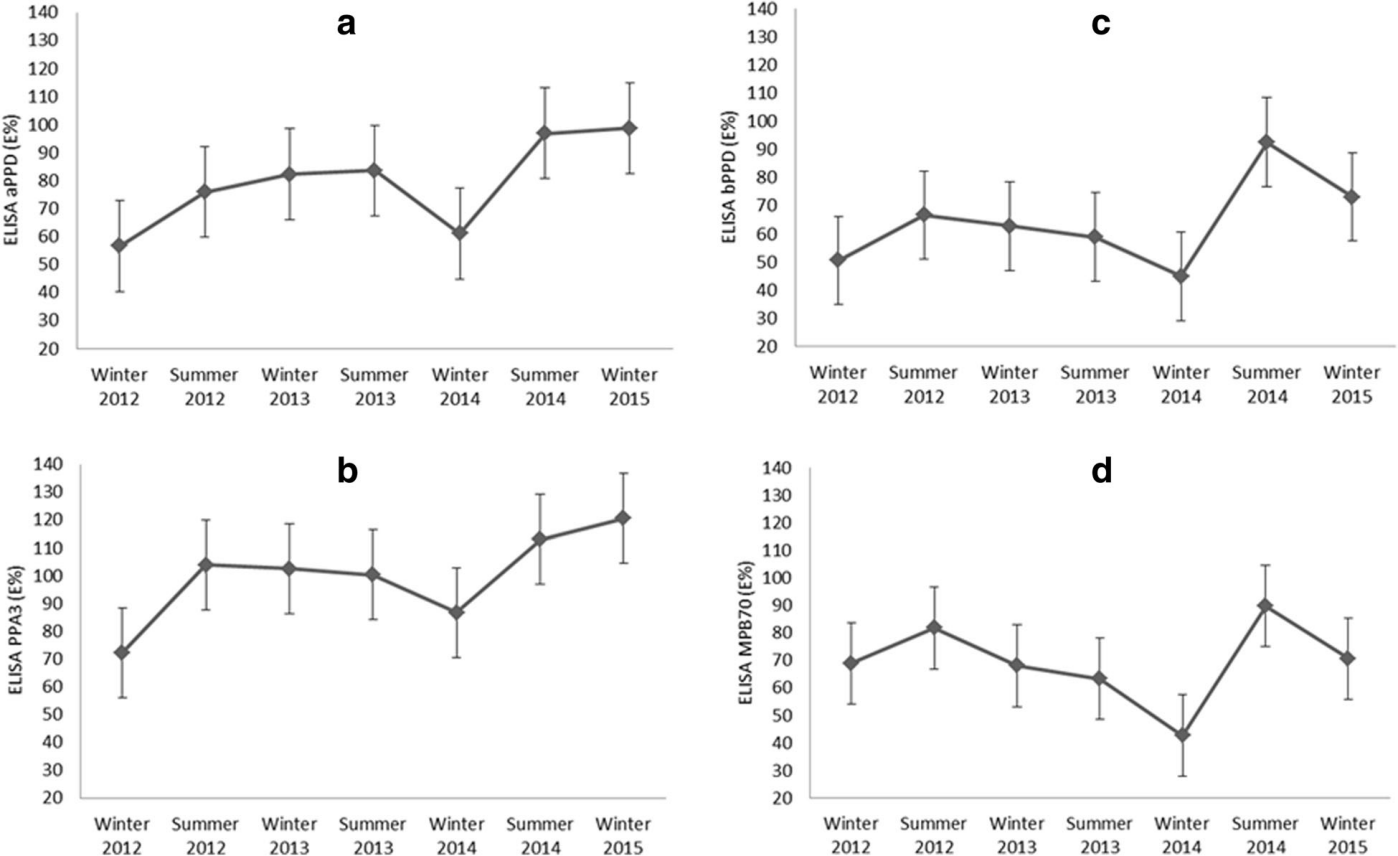
Red deer mean (±SD) seasonal (winter 2012 to winter 2015) antibody levels (in ELISA percentage, E%) against avian (**a** and **b**) and bovine (**c** and **d**) mycobacterial antigens

Figure 3 displays the mean response (in E%) to two avian and two bovine antigens, measured on serum samples taken before PPD injection for skin testing. The response to avian antigens (aPPD and PPA3; panels a and b of Fig. 3) tended to increase through time. This increase was significant for aPPD (r_s_ = 0.78, *p* < 0.05) but not for PPA3 (r_s_ = 0.64, n.s.). A total of 15 of 18 individuals (83.33 %; 14 for PPA3, 12 for aPPD) yielded a positive ELISA for antibodies against avian antigens in at least one testing round. Regarding the bovine antigens (bPPD and MPB70; panels c and d of Fig. 3), these did not increase through time (r_s_ < 0.47; n.s.). Rather, there was an increase in the ELISA response to both bovine antigens at the 6th round of testing. A total of 12 of 18 individuals (66.66 %; 12 for MPB70, 11 for bPPD) yielded a positive ELISA for antibodies against bovine antigens in at least one testing round. Animals responding to avian antigens tended to repeat and even increase their positive response through time, while responses to bovine antigens tended to be weak and sporadic, occurring more often in the 6th and 7th testing round (Additional file 2. Table S2). Only in one case, and only in one testing round, a deer hind tested positive to bovine antigens (a weak 102 % to bPPD, cut-off set at 100 %) without testing positive to avian antigens, too.

The ELISA responses to different antigens were correlated with the shape of the graphs for aPPD and bPPD was similar (r_s_ = 0.96; *p* < 0.05), while there was no correlation with the independent PHA (r_s_ < 0.51; n.s.), reflecting likely cross-reactions. In both the avian and bovine ELISAs, a marked drop was recorded at testing round 5. This drop coincided in time with a marked peak in the skin test response to PHA (Figs. 1c and 3).

**Fig. 4:**
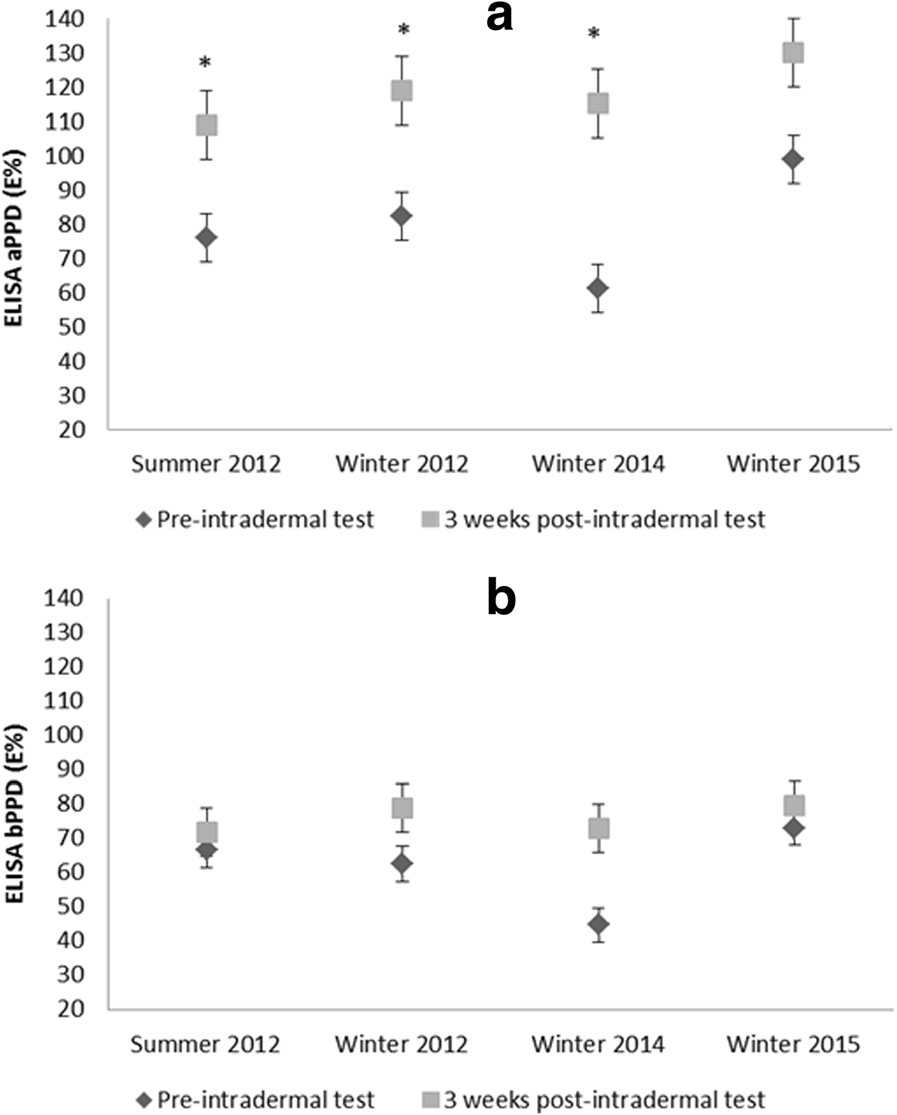
Red deer mean (±SD) antibody levels (in ELISA percentage, E%) against avian PPD (**a**) and bovine PPD (**b**) measured by ELISA immediately before and three weeks after selected skin testing rounds (*p ≤ 0.05)

Blood sera taken three weeks after selected skin testing rounds (in summer 2012, winter 2013, winter 2014 and winter 2015), allowed measuring eventual boosts in antibody levels against the antigens used for skin stimulation. Figure 4 presents the mean (±SD) response (in E%) in the production of antibodies against aPPD and bPPD before and three weeks after inoculation for each testing round (boosting effect). Antibodies against both antigens increased after skin-testing at all four times studied, i.e. the boosting effect was stable through time. This boosting effect was more evident for avian PPD than for bovine PPD (Fig. 4).

## Discussion

Based on the observed results, we rejected our initial hypothesis that repeated (six monthly) skin testing with aPPD and bPPD would cause progressive changes in skin test or ELISA responsiveness in farmed red deer. Specifically, we observed no progressive loss of the skin test response to bPPD, in contrast to previous findings in repeatedly tested cattle [12, 13]. The desensitization phenomenon described in cattle was observed in animals in which the intradermal skin test was repeated in a period of time lower than six months, which is the interval respected in this study. In addition, some studies in cattle were performed in infected animals [12, 13]. These differences may contribute to explain the results of this study.

There was also no progressive change of the boosting effect on the ELISA responses. In our study, consistent increases in response through time were only recorded for single ELISAs using avian antigens, particularly aPPD. This increase did not correlate with the skin test data, suggesting they were independent. Moreover, this increase was not recorded for the bovine antigens, suggesting that it might have been due to exposure to MAP or to other cross-reacting environmental mycobacteria in the farm. In the same way, the boosted response seen to bPPD, smaller than the one to aPPD, may represent cross reactive antibodies to shared antigens between aPPD and bPPD, or cross-reactions with environmental mycobacteria. The presence of avian and environmental mycobacteria and their potential interference in TB/PTB diagnosis in this specific deer farm had been reported earlier [5].

However, we recorded a possible temporal effect of the first skin testing round on skin-test and ELISA responsiveness in the 2nd and 3rd testing rounds. Apparently, stimulation with avian and bovine PPD during the first skin test caused an increase in the mean response to these antigens (but not with the control mitogen, PHA) in the 2nd and 3rd skin tests, and also a consistent increase in the antibodies against avian and bovine antigens in the 2nd testing round. Since our study site was a deer farm where all deer are first skin tested at the age of 8 months (1st testing round), we could not differentiate if this increased responsiveness recorded at the age of 14 months (2nd testing round) was actually an effect of the 1st skin test or due to developmental factors. However, the response to the independent mitogen PHA declined from the 1st to the 2nd round, suggesting that there was no age-related increase in the cellular responsiveness to intra-dermal antigens. An alternative or complementary explanation for this transitory increase in the skin test response to PPDs is that, as seen in cattle, the infection or exposure to *M. avium* or MAP could cause a transient increased cellular immune response [39].

At the 3 mm cut-off for aPPD and the 2 mm cut-off for bPPD, the intradermal skin test detected a high percentage of deer (72 %) as avian and as bovine positives, in at least one of the seven testing rounds. This occurred more often in the first three testing rounds (Fig. 2). The fact that we recorded a clear increase in the response to PPDs in the 2nd and 3rd testing rounds, and in some individuals even already in the first round, can lead to false-positive reactors and needs to be accounted for when interpreting skin test results in calf and yearling hinds (i.e. until their second winter or 4th testing round).

When MTC infection happens, the decrease of the cell mediated immune response may correlate with higher levels of antibodies and the development of extended TB lesions [28, 40]. In this study, the opposite situation was recorded at testing round 5, with higher skin test responses, particularly to PHA. However, as this farm was TB-free, our interpretation is that this unexpected peak was due to environmental factors. The combination of methods based on the cellular response against *M. bovis* along with serological tests may increase the chances of detection of the infectious agent and facilitate to manage TB outbreaks [32, 41]. This study showed that in TB-free red deer, there was no permanent boosting effect on serological test results after repeated tuberculin tests. As expected, the ELISA responses to different mycobacterial antigens were correlated, reflecting the likelihood of cross-reactions. Animals responding to avian antigens tended to repeat and even increase their positive response through time, suggesting true contact with avian or environmental mycobacteria. In contrast, responses to bovine antigens tended to be weak and sporadic, occurring more often in the last (6th and 7th) testing rounds, possibly as a consequence of increasing cross-reaction with avian or environmental mycobacteria.

However, re-testing for antibodies responses three weeks after each skin test helped in discarding possible cross-reactions, since the boosts were much more evident for the avian antigen (aPPD) than for the bovine antigen (bPPD) (Fig. 4). This boosting effect has a benefit by maximizing the detection of reactors through serological tests. In studies on *M. bovis* infected cattle, the injection of PPDs for skin testing boosted the responses to certain antigens (i.e. MPB83 and MPB70; [42]). In human tuberculosis, the boosting effect is maximal if the interval between the initial and second test is between 1 to 5 week [43] and is much less frequent if the interval is only 48 h [44] or more than 60 days [43], although boosting has been detected one or more years after a first negative tuberculin test [44, 45]. In experimental goat tuberculosis (*M. caprae*), it showed a similar trend as described in cattle and human tuberculosis. The sensitivity of ELISA against MPB83 increased dramatically 2 weeks after boosting with SICCT [46].

## Conclusion

In summary, repeated (every six months) administration of avian and bovine PPDs for skin testing did not result in continued sensitizing or desensitizing effects on the skin test response in red deer from a TB-free herd. Repeated skin testing had also no continued effect on serum antibody responses against avian and bovine mycobacterial antigens. The boosting effect of skin testing on antibody levels recorded three weeks later was stable. However, transitory increases in skin test responsiveness during one year (two testing rounds) and in ELISA results during six months (one testing round) cannot be discarded. The findings observed in the present work may been taken into account when designing and interpreting TB diagnosis schemes in TB-free farmed red deer and similar wildlife species.

## Additional files

Additional file 1: Table S1. ELISAs for *Mycobacterium tuberculosis* complex (MTC) and *Mycobacterium avium* paratuberculosis (MAP)-specific antibody detection in red deer (*Cervus elaphus*) (DOCX 14 kb) Additional file 2: Table S2. Individual results for red deer hinds under repeated (every 6 months) comparative tuberculin skin testing on skin test and ELISA response (in E%). In the skin test the animals were considered TB reactors if the skin fold increase to bPPD was greater than 2 mm and more than 1 mm larger than the skin fold increase to aPPD; and avian reactors were defined as those with a skin fold increase to aPPD >3 mm (all single reactors are in yellow). In the ELISA tests for antibodies against avian antigens (aPPD and PPA3) and bovine antigens (bPPD and MPB70), sera with E% values greater than 100 were considered positive (in red) (XLS 120 kb)

## Abbreviations

aPPD/bPPD: Avian/bovine purified protein derivatives
E%: ELISA percentage
ELISA: Enzyme-linked immunosorbent assay
MAP: *Mycobacterium avium paratuberculosis*
MTC: *Mycobacterium tuberculosis* complex
OD: Optical density
PBST: Phosphate buffer saline with 0.05 % Tween-20
PHA: Phytohaemagglutinin
PPA3: Paratuberculosis protoplasmatic antigen-3
PTB: Paratuberculosis
SICCT: Single intradermal comparative cervical tuberculin test
TB: Tuberculosis
