# Supplementary material for: Effects of repeated comparative intradermal tuberculin testing on test results: a longitudinal study in TB-free red deer
Source: BMC Vet Res. 2016 Sep 5;12(1):184. doi: 10.1186/s12917-016-0825-2 (PMC5011987; doi:10.1186/s12917-016-0825-2)
Supplement: Additional file 1: Table S1. — ELISAs for Mycobacterium tuberculosis complex (MTC) and Mycobacterium avium paratuberculosis (MAP)-specific antibody detection in red deer (Cervus elaphus) (DOCX 14 kb) [file 12917_2016_825_MOESM1_ESM.docx]

| **Assay test** | **Antigens** | **Sensitivity (%)** | **Specificity (%)** | **Reference** |
| --- | --- | --- | --- | --- |
| CervidTB  STAT-PAK  DPP VetTB  MAPIA  ELISA | ESAT-6/CFP-10, MPB83  ESAT-6/CFP-10, MPB83  ESAT-6, CFP10, MPB59, MPB64, MPB70, MPB83, the 16-kDa protein, the 38-kDa protein, two fusion proteins comprising CFP10/ESAT-6 and the 16-kDa protein/MPB83,  two native antigens, bovine PPD and *M. bovis* culture filtrate.  bPPD  MPB83  Extracted *M. bovis* surface antigen  Johnin PPD (jPPD)  PpAg | 75.0, 82.0  75.0, 79.0  82.0  72.7  86.7  50.0, 84.0  91.0 | 83.8, 93.0  91.4, 98.0  93.0, 99.5  88.0 | Buddle et al 2010, Waters et al 2011  Buddle et al 2010, Waters et al 2011  Buddle et al 2010, Waters et al 2011  García-Bocanegra et al 2012  Wadhwa et al 2013  Crawford et al 2006, Griffin et al 2005  Griffin et al 2005 |
